# *In Vivo* Time-gated Fluorescence Imaging with Biodegradable Luminescent Porous Silicon Nanoparticles

**DOI:** 10.1038/ncomms3326

**Published:** 2013

**Authors:** Luo Gu, David J. Hall, Zhengtao Qin, Emily Anglin, Jinmyoung Joo, David J. Mooney, Stephen B. Howell, Michael J. Sailor

**Affiliations:** 1 Department of Chemistry and Biochemistry, University of California, San Diego, La Jolla, California 92093, USA; 2 School of Engineering and Applied Sciences; 3 Wyss Institute for Biologically Inspired Engineering, Harvard University, Cambridge, Massachusetts 02138; 4 Department of Radiology, University of California, San Diego, La Jolla, California 92093, USA; 5 Moores Cancer Center, University of California, San Diego, La Jolla, California 92093, USA; 6 Department of Medicine, University of California, San Diego, La Jolla, California 92093, USA

## Abstract

Fluorescence imaging is one of the most versatile and widely used visualization methods in biomedical research. However, tissue autofluorescence is a major obstacle confounding interpretation of *in vivo* fluorescence images. The unusually long emission lifetime (5-13 μs) of photoluminescent porous silicon nanoparticles can allow the time-gated imaging of tissues *in vivo*, completely eliminating shorter-lived (< 10 ns) emission signals from organic chromophores or tissue autofluorescence.Here, using a conventional animal imaging system not optimized for such long-lived excited states, we demonstrate improvement of signal to background contrast ratio by > 50-fold *in vitro* and by > 20-fold *in vivo* when imaging porous silicon nanoparticles. Time-gated imaging of porous silicon nanoparticles accumulated in a human ovarian cancer xenograft following intravenous injection is demonstrated in a live mouse. The potential for multiplexing of images in the time domain by using separate porous silicon nanoparticles engineered with different excited state lifetimes is discussed.

Fluorescence imaging has become a dominant *in vitro* and *in vivo* visualization method in biomedical research due to its high sensitivity, its high spatial resolution, and its ease of use^1, 2^. In vivo imaging of exogenous fluorescent probes that target diseased tissues has also shown promising results in clinical settings, such as the early detection of breast cancer, the outlining of tumor margins during surgery, and endoscopic diagnosis of cancer micrometastasis^1, 3-5^. However, the method is limited by tissue attenuation (scattering and absorption of the excitation or the emission light) and by tissue autofluorescence^6, 7^. To minimize tissue attenuation effects, researchers have concentrated on near-infrared (NIR) fluorophores that are excited and emit in the spectral window between wavelengths of 650 - 950 nm^8-11^. However, tissue autofluorescence still produces a substantial background signal in this spectral range that severely limits the quality of images, especially when very low concentrations of the fluorescent probe accumulate at the target site^12^.

The endogenous fluorophores responsible for tissue autofluorescence have decay lifetimes of ~ 1 - 10 ns, depending on the type of tissue^7^. It has been proposed that late time-gating (i.e., capturing the signal at a delayed time after excitation) could be used to image molecular or quantum dot imaging probes in the presence of this interference^13-18^. However, there is a lack of biocompatible NIR fluorophores with fluorescence lifetimes significantly greater than 1-10 ns, due to the quantum mechanical selection rules associated with organic molecules or direct gap semiconductors^7, 19^.

Electrochemically etched porous silicon has shown considerable potential for *in vivo* applications due to its biodegradability, its low toxicity, its large specific capacity for drug loading, and its intrinsic photoluminescence^20-27^. Silicon is an indirect gap semiconductor, which gives it a much longer-lived excited state than direct gap semiconductors such as CdS or CdSe^28^. This property translates to the scale of quantum dots, and nanoparticles derived from silicon tend to display radiative lifetimes on the order of 100 ns to several microseconds^29-31^. In contrast, CdS and CdSe excited states decay on much shorter timescales of a few nanoseconds to several tens of nanoseconds^32^. We recently demonstrated that NIR luminescent nanoparticles made of porous silicon (LPSiNPs) can be prepared that are biocompatible, have low systemic toxicity, and that accumulate in tumors *in vivo* and then degrade into components cleared by the kidneys^33^.

Here, we show that the emission lifetime of these nanoparticles is sufficiently long (5 -13 μs) to permit late time-gated imaging.The persistent emission from LPSiNPs is well differentiated in the time domain from signals associated with tissue autofluorescence or interfering organic chromophores. Delayed (late time-gated) images acquired18 ns after excitation show clear signals from LPSiNPs, while the background fluorescence has disappeared due to its much shorter lifetime. An example of the utility of the method is demonstrated in the imaging of a human ovarian cancer xenograft in a mouse model, using LPSiNP probes introduced via intravenous injection.

## Results

### Long emission lifetime of LPSiNPs

**Figure 1:**
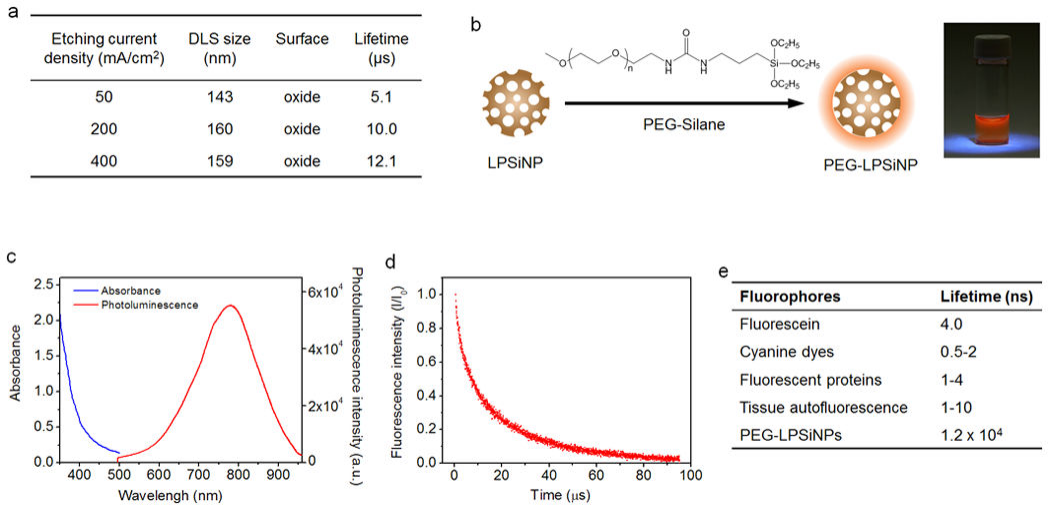
Characterization of polyethylene glycol-conjugated luminescent porous silicon nanoparticles **a**, Hydrodynamic diameter, surface chemistry, and emission lifetime of LPSiNPs prepared at the indicated etch current densities. **b**, Schematic diagram depicting the PEG surface chemistry on the LPSiNPs. Photograph of PEG-LPSiNPs in water, obtained under UV light illumination. **c**, Absorbance and steady-state photoluminescence spectrum of PEG-LPSiNPs (λ_ex_ = 370 nm). **d**, Normalized photoluminescence intensity-time trace for PEG-LPSiNPs after pulsed excitation (λ_ex_ = 456 nm, λ_em_ = 650 nm, 22 °C). **e**, Fluorescence lifetimes of commonly used fluorophores and tissue autofluorescence^7, 42^.

LPSiNPs were prepared by electrochemical etching of single-crystal Si in HF-containing electrolyte, followed by lifting-off of the porous layer, ultrasonic fracture, filtration of the resulting nanoparticles through a 0.22 μm filter membrane, and finally activation of photoluminescence by treatment in an aqueous solution following the published procedure^33^. The emission lifetime of the nanoparticles was controlled by adjusting the current density used in the electrochemical etch (Fig. 1a and Supplementary Figs S1 and S2) and by post-etching chemical treatments (Supplementary Table S1). For the time-gated fluorescence imaging study presented here, the LPSiNPs were prepared using electrochemical etching at a current density of 200 mA/cm^2^ with a two-week aqueous activation treatment. The nanoparticles were coated with 5 kDa polyethylene glycol (PEG) by reaction with a PEG-silane (PEG-LPSiNP, Fig. 1b). The mean hydrodynamic diameter of the PEG-LPSiNP formulation (measured by dynamic light scattering, DLS) was 168 nm, consistent with transmission electron microscopy (TEM) measurements (Supplementary Fig. S3). The photoluminescence spectrum from the PEG-LPSiNPs (λ _ex_ = 370 nm) appeared at wavelengths between 600 and 900 nm (Fig. 1c), and the decay lifetime was 12 μs (λ _em_ = 650 nm, 22 °C). This lifetime is >1000 times larger than that typical of tissue autofluorescence or common fluorescent imaging dyes (Fig. 1, d and e; Supplementary Fig. S4, S5).

**Figure 2:**
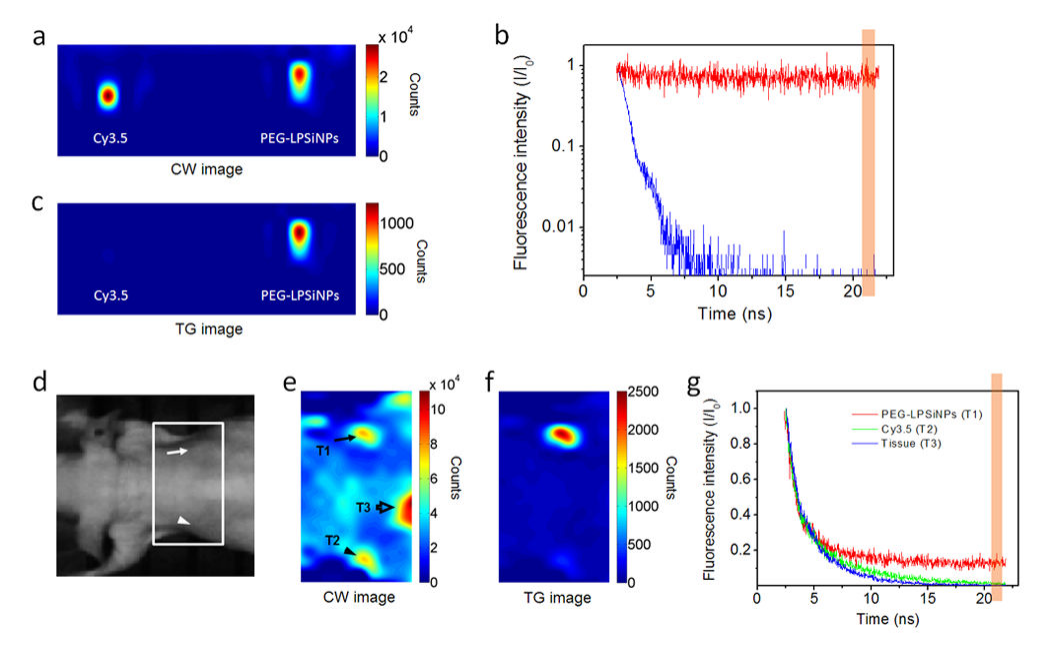
Comparison of time-domain fluorescence characteristics of PEG-LPSiNPs and the common imaging fluorophore Cy3.5 **a**, Steady-state, or continuous wave (CW) fluorescence images of Cy3.5 (0.01 mg/mL) and PEG-LPSiNPs (0.1 mg/mL) samples in microtubes (λ_ex_ = 470 nm, λ_em_ = 590 nm longpass). **b**, Normalized intensity decay of the fluorescence/photoluminescence signals from the Cy3.5 and PEG-LPSiNPs samples shown in (**a**) as a function of time after excitation pulse. The vertical orange bar depicts the time window used in the time-gating algorithm (20.5-21.5 ns of the 25 ns imaging window, which is 18-19 ns after excitation pulse) to obtain time-gated (TG) fluorescence images; i.e., the images depicted as “TG” represent fluorescence intensity integrated between 18-19 ns after the excitation pulse. **c**, TG image of the same microtubes in (**a**). The image of the Cy3.5 sample almost completely disappears due to its short-lived emission. **d**, Bright field image of a nude mouse injected subcutaneously with PEG-LPSiNPs (20 μL, 0.5 mg/mL) and Cy3.5 (20 μL, 0.02 mg/mL). The arrow indicates the injection site of PEG-LPSiNPs, and the arrowhead indicates the injection site of Cy3.5. **e**, CW fluorescence image of the region of the mouse indicated by the white box in (**d**), obtained with the Optix imaging system. Regions identified as T1, T2, and T3 are the injection site of PEG-LPSiNPs, the injection site of Cy3.5, and the abdomen, respectively. **f**, TG image of the same region shown in (**e**). Signals from the Cy3.5 dye (T2) and from tissue autofluorescence (T3) almost disappear. The intensity ratio of PEG-LPSiNPs to tissue autofluorescence was calculated by comparing the CW to the TG signal intensities of PEG-LPSiNPs (T1, arrow) and tissue autofluorescence (T3, hollow arrow) as indicated in (**e**). **g**, Normalized intensity decay of the fluorescence signals from T1, T2, and T3 in (**e**), as indicated. The vertical orange bar depicts the time window used in the time-gating algorithm for (**f**).

We first tested whether the long-lived photoluminescence from PEG-LPSiNPs could be separated from the fluorescence of conventional fluorophores in the time domain. To demonstrate the potential for practical applications, we used a commercial *in vivo* imaging system (eXplore Optix, ART Inc.). This instrument uses pulsed laser diodes and time-correlated single photon counting (TCSPC) to quantify fluorescence intensity and lifetime^34^. When imaged using a 470 nm excitation laser and a 590 nm longpass emission filter, the intensity of fluorescence from an aqueous solution 10 μg/mL of the common imaging dye Cy3.5 was comparable to the intensity of photoluminescence from an aqueous suspension 100 μg/mL in PEG-LPSiNPs (Fig. 2a). The laser we used operates at a repetition rate of 40 MHz, corresponding to a time window between pulses of 25 ns. The duration of an individual pulse from the laser is < 0.1 ns. Although the Cy3.5 sample and the PEG-LPSiNP sample showed similar photoluminescence intensity when integrated over the entire 25 ns window (Fig. 2a), temporally integrating a 1 nanosecond gate of the signal between 20.5 and 21.5 ns of the imaging window (18 and 19 ns post-pulse after correcting for synchronization) yielded a strong signal from the PEG-LPSiNP sample (50 times greater than background), while the signal from the Cy3.5 sample was at or below the noise level of the instrument (Fig. 2, b and c). This improvement in image contrast is due to the rapid decay of fluorescence from the organic fluorophore (0.6 ns lifetime); the photoluminescence signal from the nanoparticle is essentially constant for the duration of the 25 ns period between pulses (Fig. 2b). As a result, only the PEG-LPSiNP sample was visible under late time-gating conditions (Fig. 2c).

### *In vivo* time-gated imaging with PEG-LPSiNPs

We next investigated the possibility of eliminating tissue autofluorescence or interference from exogenous fluorophores when imaging PEG-LPSiNPs *in vivo*. A 20 μL aliquot of PEG-LPSiNPs (0.5 mg/mL) and a 20 μL aliquot of Cy3.5 (0.02 mg/mL) in normal saline were injected subcutaneously into the right and left shoulder of a nude mouse, respectively (Fig. 2d). When imaged under pseudo continuous wave (CW), or steady-state conditions (i.e., no time gating), the two injection points had intensities comparable to the brighter autofluorescent tissues (Fig. 2e). However, the intensity-time decay curves of the relevant regions revealed a distinct persistence of signal only for the PEG-LPSiNPs (Fig. 2g). As shown in Fig 2g, the fast decay component (dominant at times <5 ns) from ubiquitous tissue auto fluorescence appeared similar in all the body regions imaged: the PEG-LPSiNP injection site (T1), the Cy3.5 injection site (T2), and the background tissue region (T3). Only at the site of PEG-LPSiNP injection was residual photoluminescence intensity observed at times >5ns, consistent with the longer lifetime of PEG-LPSiNPs. As predicted by the *in vitro* data of Fig. 2b, the signal from PEG-LPSiNPs was relatively constant between ~10 and 22 ns. To confirm this persistent signal at the injection site arose from PEG-LPSiNPs, we measured the full decay *in vivo* on a 100 μs time scale (Supplementary Fig. S5). In agreement with the results in Fig. 2g, both control tissue and tissue injected with PEG-LPSiNPs showed a prompt decay from tissue autofluorescence (<5ns). However, only the tissue containing PEG-LPSiNPs showed a slow photoluminescence decay component, consistent with the *in vitro* measurements of PEG-LPSiNPs (Fig. 1d, Supplementary Fig. S5). Due to the slower radiative decay rate and low injection dose, this long-lived signal is weaker than the signals from Cy3.5 or from tissue autofluorescence, though it is easy to discern in the time-resolved data (Fig. 2g, T1). Application of the late time-gating algorithm (1 ns gate, 18 ns post-excitation) revealed a distinct spot in the PEG-LPSiNP injection point (T1), with negligible intensity from the Cy3.5 injection or from background tissue autofluorescence (Fig. 2f). Simple analysis of pixel intensities revealed a 20-fold increase in image contrast with the time-gated (TG) image relative to the CW image (Fig. 2, e and f). It is possible that excitation crossover (bleed-through of the excitation source scattered from the tissues), a common problem for *in vivo* fluorescence imaging, may also contribute to the background signals in CW fluorescence images. Since the pulse width of the excitation laser was <0.1 ns, the time-gating method used here was able to remove this potential interference.

### PEG-LPSiNPs are distinguishable from fluorescent proteins

Genetically expressed fluorophores such as fluorescent proteins are widely used in biomedical research, so we next tested time-gated imaging of PEG-LPSiNPs in the human ovarian carcinoma 2008 cell line expressing the red fluorescent protein mCherry (2008-mCherry, Supplementary Fig. S6a). The fluorescence lifetime of mCherry is ~1.4 ns^35^, (verified in Supplementary Fig. S6b). In vitro imaging of PEG-LPSiNPs and 2008-mCherry cells showed that the signal from the nanoparticles was readily distinguishable using late time-gated imaging (Supplementary Fig.S6, c and d).

**Figure 3:**
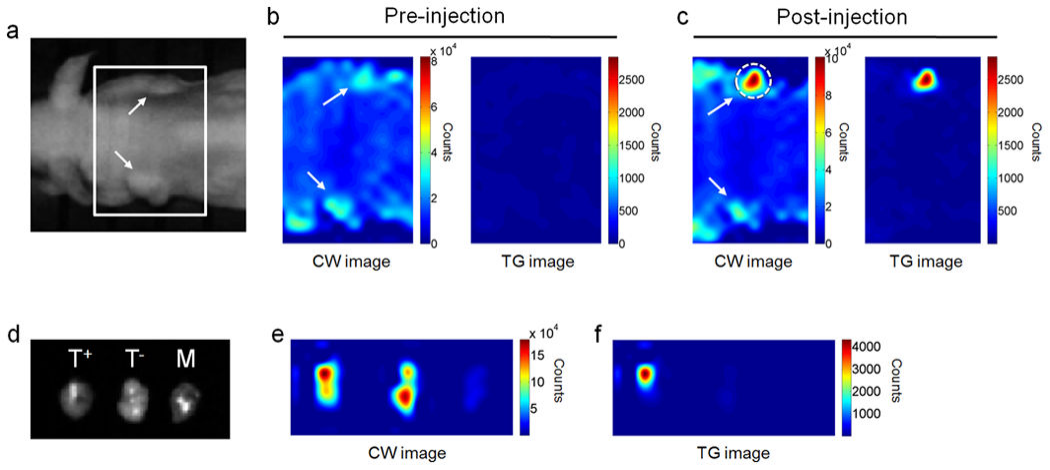
Time-gated fluorescence images comparing PEG-LPSiNP with fluorescent protein mCherry in a nude mouse bearing mCherry-expressing tumors **a**, Bright field photograph of mouse containing two tumors, one on each shoulder. Tumors are indicated with white arrows. **b**, CW and TG (as defined in Fig. 2) fluorescence images of the tumors, showing low differentiation of the expressed mCherry and tissue autofluorescence in the time-domain. **c**, CW and TG fluorescence images of the tumors after injection of PEG-LPSiNPs (50 μL, 0.2 mg/mL) into the right shoulder tumor (on the top of the image, indicated with dashed white circle). **d**, Ex vivo bright field image of the PEG-LPSiNP-injected tumor (T^+^), a control tumor (T^-^), and muscle tissue excised from the animal post-injection. **e**, CW fluorescence image of the excised tissues in (**d**). **f**, TG fluorescence image of the tissues in (**d**).

The PEG-LPSiNPs were also readily distinguishable from mCherry *in vivo*. A nude mouse bearing a 2008-mCherry tumor xenograft on each shoulder was imaged. The CW image displayed fluorescence from both tumors as well as strong tissue autofluorescence (Fig. 3, a and b). The fluorescence signal from mCherry could not be distinguished from tissue autofluorescence due to their similar short fluorescence lifetimes (Fig. 3b). PEG-LPSiNPs (50 μL, 0.2 mg/mL) were then injected into the tumor on the right shoulder of the mouse, and the mouse was imaged again under CW conditions. The tumor injected with nanoparticles was brighter than the control tumor in the CW image, but tissue autofluorescence and the signal from the control tumor were still clearly visible (Fig. 3c). In contrast, signals from both the control tumor and tissue autofluorescence were completely eliminated in the time-gated image (Fig. 3c). *Ex vivo* fluorescence images confirmed the presence of PEG-LPSiNPs in the tumor, and they demonstrated the effectiveness of time-gated imaging of PEG-LPSiNPs in *ex vivo* tissues (Fig. 3, d-f).

### Time-gated imaging of tumors with PEG-LPSiNPs

**Figure 4:**
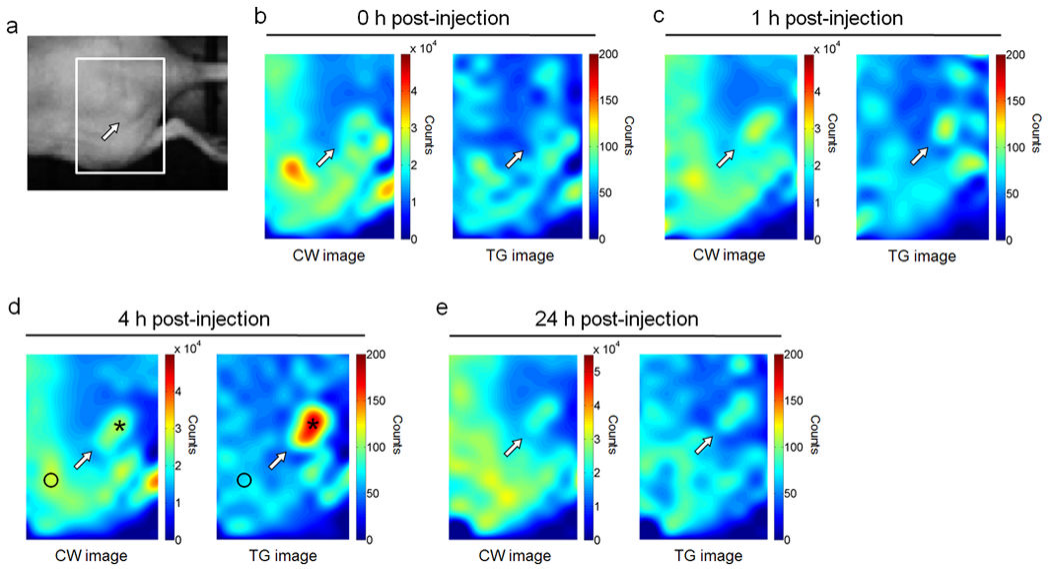
Time-gated fluorescence images of mouse bearing SKOV3 xenograft tumor after IV injection of PEG-LPSiNPs **a**, Bright field image of a nude mouse bearing a tumor at the flank. The arrow indicates the site of the tumor. **b-e**, CW and TG (as defined in Fig. 2)fluorescence images of the region indicated with the white box in (**a**) immediately (**b**), 1h (**c**), 4 h (**d**), or 24 h (**e**) post-injection of PEG-LPSiNPs (10 mg/kg body weight). The signal to background (tissue autofluorescence) ratio described in the text was calculated by comparing signal intensities at the sites indicated by the black asterisk (PEG-LPSiNPs) and the black circle (tissue autofluorescence) as indicated in (**d**) in both CW and TG images.

Finally, we evaluated the potential of imaging tumors using intravenously injected circulating PEG-LPSiNPs as the photoluminescent probe. Intravenously injected nanoparticles can passively accumulate in tumor tissues due to the enhanced permeability and retention (EPR) effect^36-38^. However, the efficiency of this process is low and generally only a small fraction of injected nanoparticles accumulates in a tumor^39, 40^. This creates a challenge for *in vivo* optical imaging because tissue autofluorescence can overwhelm the signals from the fluorescent probe. To evaluate the ability of late time-gated PEG-LPSiNPs to remove tissue autofluorescence in this situation, PEG-LPSiNPs (10 mg/kg) were injected intravenously into a nude mouse bearing a human ovarian carcinoma SKOV3 xenograft tumor (Fig. 4a). Fluorescent images of the mouse were intermittently acquired over a 24 h time period post-injection. No signal from the nanoparticles was observed in either the CW or the TG images immediately after injection because very few nanoparticles had accumulated in the tumor tissue (Fig. 4b). In addition, the lifetime of autofluorescence measured from the xenograft tumor was 2.2 ns, similar to the lifetime of the autofluorescence from healthy tissues measured in the vicinity of the tumor (2.3 ns). Therefore, no tumor contrast was detected in the TG image if the long-lived photoluminescent PEG-LPSiNP probe was not present. Weak photoluminescence was detected in the tumor 1 h after injection. However, the signal from the nanoparticles was too weak to be differentiated from tissue autofluorescence even with time-gating (Fig. 4c). As time progressed more PEG-LPSiNPs accumulated at the tumor, and the TG image obtained 4 h post-injection clearly revealed the site of the tumor (Fig. 4d). No contrast between tumor and normal tissue was observed in the CW image due to the pronounced autofluorescence signals. The intensity ratio between the tumor and normal tissues increased from ~1 in the CW image to 3 in the TD image (Fig. 4d). The signal from the tumor then decreased substantially 24 h post-injection (Fig. 4e) as the nanoparticles degraded and cleared from the host^33^. *Ex vivo* fluorescence images indicated that a small, but detectable quantity of PEG-LPSiNPs remained in the tumor 24 h post-injection (Supplementary Fig. S7).

## Discussion

Although the commercial time-domain imager used in the present study to identify and track the silicon-based nanoparticles has very good (sub-nanosecond) time resolution and excellent detector sensitivity, it is limited in the delay time that can be applied due to the repetition rate of the pulsed laser. A less sophisticated imaging system, with an ability to gate at a modest (>50 ns or even longer) delay time, is expected to yield even better image quality from PEG-LPSiNPs due to the long (microseconds) emission lifetime of this probe (Fig. 1d, Supplementary Figs S4 and S5). For example, given two emitters with the same steady-state emission intensity—a long-lived emitter such as porous Si (12 μs lifetime, 10% quantum yield) and a prompt-emitting fluorophore such as Cy3.5 (0.6 ns lifetime, 15% quantum yield)—the time-gated (1 ns window, delay of 18 ns) emission intensity of the porous Si emitter is theoretically >10^8^ times larger than the prompt emitter. This calculation assumes single-exponential decays and unitary instrument response functions. The theoretical contrast ratio rises very quickly with delay time; if imaged at a delay of 50 ns, it is >10^31^. Even if the porous Si probe is present at a concentration a million fold lower than the prompt-emitting fluorophores, the theoretical contrast ratio at 50 ns post-excitation is >10^25^. The potential sensitivity improvement obtained with later time-gating is especially helpful when a low dose of imaging probe is required.

Typically in time resolved fluorescence imaging one must contend with temporally overlapping fluorescence decays from different fluorophores and unmix these signals based on lifetime contrast using model fitting (e.g. multiexponential fits). However, late time-gated imaging using long-lifetime LPSiNPs avoids the temporal overlap of confounding tissue autofluorescence signals, eliminating the need to measure the complete fluorescence decay and unmix the signals. This approach provides a means of improving detection sensitivity and image contrast without sacrificing image resolution or generating false readings due to potential errors in model assumptions.

The wavelength distribution of the NIR emission spectrum of LPSiNPs is broader than many fluorescent probes, which places a limit on theuse of LPSiNPs for multicolor imaging in the spectral domain. However, in contrast to conventional molecular probes, the decay lifetime of LPSiNPs can be readily tuned without changing the chemical nature of the probe, using a mild aqueous treatment (Supplementary Table S1). This provides the possibility of multiplex imaging in the time domain using multiple nanoparticle probes and various time gates. Furthermore, the slow change in emission lifetime that occurs upon degradation in aqueous media may allow the nanoparticle probe to report on its age in the system. In addition to their ability to provide high contrast images by time gating, the low toxicity and biodegradable characteristics of LPSiNPs overcomes the environmental and safety disadvantages of cytotoxic or non-biodegradable fluorescent probes currently used for *in vitro* or *in vivo* imaging.

## Methods

### Preparation of PEG-LPSiNPs

Luminescent porous silicon nanoparticles (LPSiNPs) were prepared following a previously described method^33, 41^: (100)-oriented p-type single-crystal Si wafers (0.8-1.1 mΩ-cm resistivity, obtained from Siltronix, Inc.) were electrochemically etched by application of a constant current density of 200 mA/cm^2^ for 150 s in an electrolyte containing aqueous 48% hydrofluoric acid and ethanol in a 3:1 ratio. The resulting porous Si films were lifted from the Si substrate by application of a current pulse of 4 mA/cm^2^ for 250 s in a solution containing 3.3% (by volume) of 48% aqueous HF in ethanol. The porous Si film was fractured by ultrasound and filtered through a 0.22 μm membrane. Finally, the nanoparticles were mildly oxidized by soaking in deionized water for 2 weeks to activate photoluminescence. The activated nanoparticles were rinsed with deionized water 3 times by centrifugation. LPSiNP samples were prepared at the following etching current densities: 50 mA/cm^2^ for 300 s, 200 mA/cm^2^ for 150 s, or 400 mA/cm^2^ for 150 s. The activation step involved soaking of the LPSiNPs in deionized water for various periods of time: 2 weeks, 6 weeks, or 10 weeks. To prepare PEG-LPSiNPs, a 0.6 mL aliquot of an ethanolic dispersion (0.5 mg/mL LPSiNPs) was mixed with a 0.5 mL aliquot of 6 mg/mL mPEG-Silane (MW 5k, Laysan Bio, Inc.) in ethanol. The mixture was stirred for 16 h at room temperature. The nanoparticles were rinsed 3 times with ethanol and then 3 times with water. The samples were collected by centrifugation between each rinsing step.

### Property measurements of LPSiNPs

Dynamic light scattering (Zetasizer Nano ZS90, Malvern Instruments) was used to determine the hydrodynamic size of the nanoparticles. Transmission electron microscope (TEM) images were obtained with a FEI Tecnai G^2^ Sphera. Scanning electron microscope (SEM) images were obtained using a Philips XL30 field emission ESEM operating in secondary electron emission mode. The photoluminescence (λ_ex_ = 370 nm, 460 nm long pass emission filter) spectra of LPSiNPs or PEG-LPSiNPs were obtained using a Princeton Instruments/Acton spectrometer fitted with a liquid nitrogen-cooled silicon charge-coupled device (CCD) detector. The photoluminescence decay data for aqueous dispersions of nanoparticles (in water or in tissue homogenates) were acquired using a Horiba Scientific FluoroLog-3 spectrofluorometer using a time correlated single photon counting (TCSPC) method. A 456 nm NanoLED (Horiba Scientific) at 10 kHz repetition rate was used as the excitation source. The signal was collected at 650 or 700 nm at 22 °C. Although the decay curves for the PSiNPs were nonexponential, average decay lifetime is reported as the time at which the photoluminescence intensity of the nanoparticles decreased to l/*e* of the initial value after excitation. For the *in vivo* photoluminescence decays obtained on a longer (microseconds) timescale (Supplementary Fig. S5), spectra were acquired using a home-built system. A 10 Hz pulsed Nd:YAG laser (excitation at 355 nm, Quantel) was used as the source, and the PL signal was recorded with a high speed Si photo detector (DET110, Thorlabs) coupled to a 400 nm longpass filter and an oscilloscope (DPO3054, Tektronix).

### Cell lines and mice

Two human ovarian carcinoma cell lines SKOV3 and 2008 were used in this study. 2008 cells were transduced with lentiviral vectors containing the red fluorescent protein mCherry sequence (pLVX-mCherry, Clontech). The cells expressing mCherry (2008-mCherry) were sorted by flow cytometry and maintained in RPMI-1640 medium with 10% fetal calf serum (Invitrogen). Female nu/nu nude mice (Charles River) were maintained in specific pathogen-free facilities at the University of California, San Diego. Animal protocols were approved by the Institutional Animal Care and Use Committee.

### *In vitro* and *in vivo* fluorescence imaging

A time-domain fluorescence imaging system eXplore Optix (ART Advanced Research Technologies, Inc.) was used to image fluorophore solutions and cell suspensions *in vitro*, and mice *in vivo*. A 470 nm laser with 40 MHz repetition rate was used as the excitation source and a 590 nm longpass filter was used as the emission filter for all imaging experiments.

For *in vitro* comparison of PEG-LPSiNPs and Cy3.5, an aliquot of PEG-LPSiNPs in aqueous dispersion (50 μL, 0.1 mg/mL) was placed in a microtube, and an aliquot of Cy3.5 NHS ester (GE Healthcare) in aqueous solution (50 μL, 0.01 mg/mL) was placed in a separate microtube. The microtubes were placed next to each other and imaged at the same time with the Optix imaging system. For *in vivo* imaging, PEG-LPSiNPs (20 μL, 0.5 mg/mL) and Cy3.5 (20 μL, 0.02 mg/mL) were injected subcutaneously into the right and left shoulder of a nude mouse, respectively. The mouse was imaged with the Optix imaging system immediately after injection.

For *in vitro* comparison of PEG-LPSiNPs with mCherry, aliquots of PEG-LPSiNPs (20 μL, 0.2 mg/mL) and 2008-mCherry cell suspension (20 μL, ~1 million cells) were placed in separate microtubes and imaged at the same time with the Optix imaging system. For *in vivo* imaging, a nude mouse bearing a 2008-mCherry tumor (~0.7 cm) on each side of the shoulder was used. An aliquot of PEG-LPSiNPs dispersion (50 μL, 0.2 mg/mL) was injected into the tumor on the right shoulder of the mouse, and the mouse was imaged with the Optix imaging system immediately after injection.

Nude mice bearing a SKOV3 tumor (~0.5 cm, left flank) were used for the *in vivo* tumor imaging study. A PEG-LPSiNPs saline dispersion was injected intravenously into the mouse at a dose of 10 mg/kg body mass through the tail vein. The mouse was imaged at several different time points for 24 h post-injection. The tumor and muscle in the vicinity of the tumor were harvested and imaged 24 h after injection.

### Data analysis and time gating

The Optix imaging system data were analyzed using OptiView (ART Advanced Research Technologies, Inc.) to calculate the in vivo decay lifetimes of the fluorophores or tissue autofluorescence. Continuous wave (CW) fluorescence images were obtained by reconstructing the photoluminescent signal collected within the full 25 ns imaging time-window from the data using MATLAB (MathWorks, Inc.). Time-gated (TG) fluorescence images were generated by extracting and temporally integrating the photoluminescent signal collected between 20.5 and 21.5 ns of the imaging time-window (18-19 ns after the excitation pulse) from the data using a subroutine in the computer program MATLAB.

## Supplementary Material

1
