# Supplementary material for: In Vivo Time-gated Fluorescence Imaging with Biodegradable Luminescent Porous Silicon Nanoparticles
Source: Nat Commun. Author manuscript; Available in PMC 2014 Sep 4. (PMC4154512; doi:10.1038/ncomms3326)
Supplement: 1 [file NIHMS508100-supplement-1.pdf]

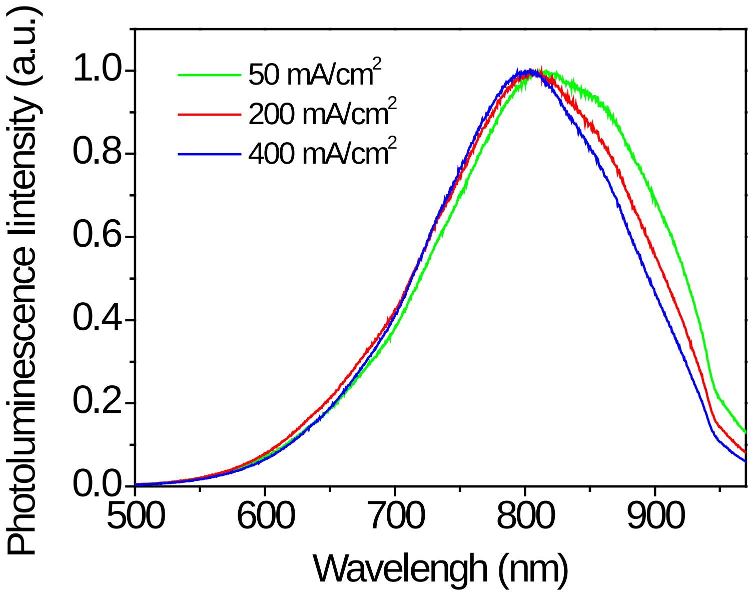

Supplementary Figure S1 | Steady-state photoluminescence (PL) spectra of LPSiNPs in deionized water, prepared at etching current densities of 50 mA/cm^2, 200 mA/cm^2, or 400 mA/cm^2 as indicated. All samples were activated in deionized water for 2 weeks prior to data acquisition. Excitation wavelength was λ_{ex}= 370 nm.

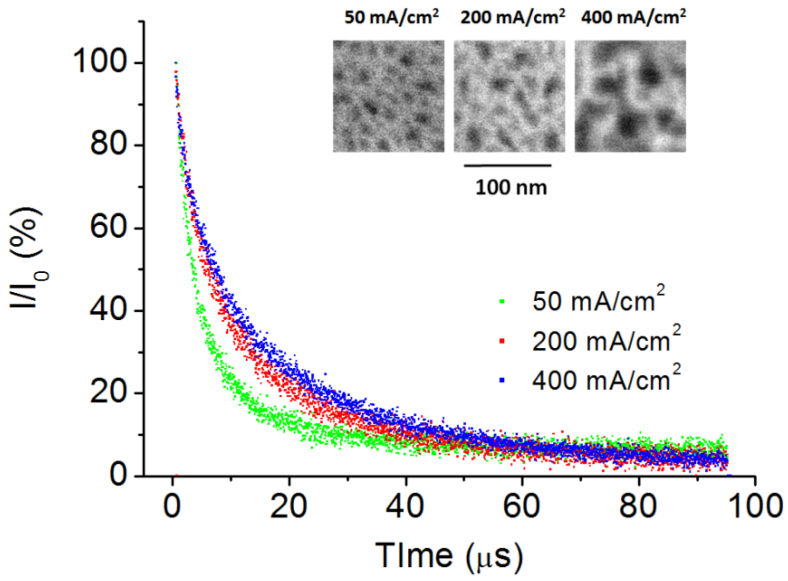

Supplementary Figure S2 | Photoluminescence decay curves for LPSiNPs prepared at etching current densities of 50 mA/cm^2, 200 mA/cm^2, or 400 mA/cm^2, as indicated. Inset shows scanning electron microscope (SEM) images of the porous nanostructures of the porous Si films prior to nanoparticle formation.

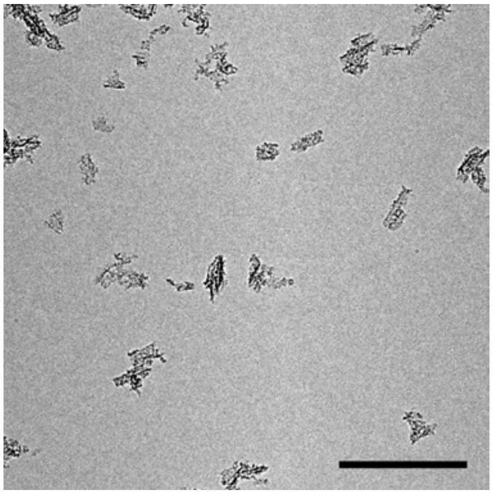

Supplementary Figure S3 | Transmission electron microscope image of polyethylene glycol coated LPSiNPs (PEG-LPSiNPs). Scale bar is 500 nm.

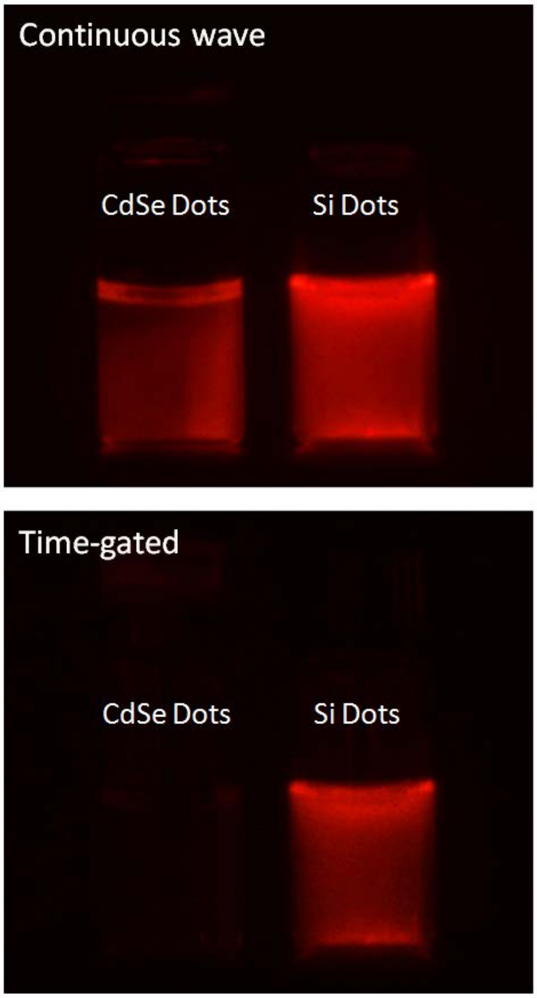

Supplementary Figure S4 | Steady-state (top) and time-gated (bottom) fluorescence images comparing water dispersions of PEG-LPSiNPs with commercial CdSe:ZnS core-shell quantum dots in cuvettes, obtained under UV light illumination (360 nm LEDs, Nichia, inc.). Images obtained with an ANDOR, inc. time gated digital camera. The time-gated photoluminescence image was obtained with a 2 microsecond delay. On left are conventional CdSe material ("CdSe Dots"), on right are the porous Si nanoparticles ("Si Dots"). Images are artificially colored orange to match the visual appearance of both nanoparticle samples.

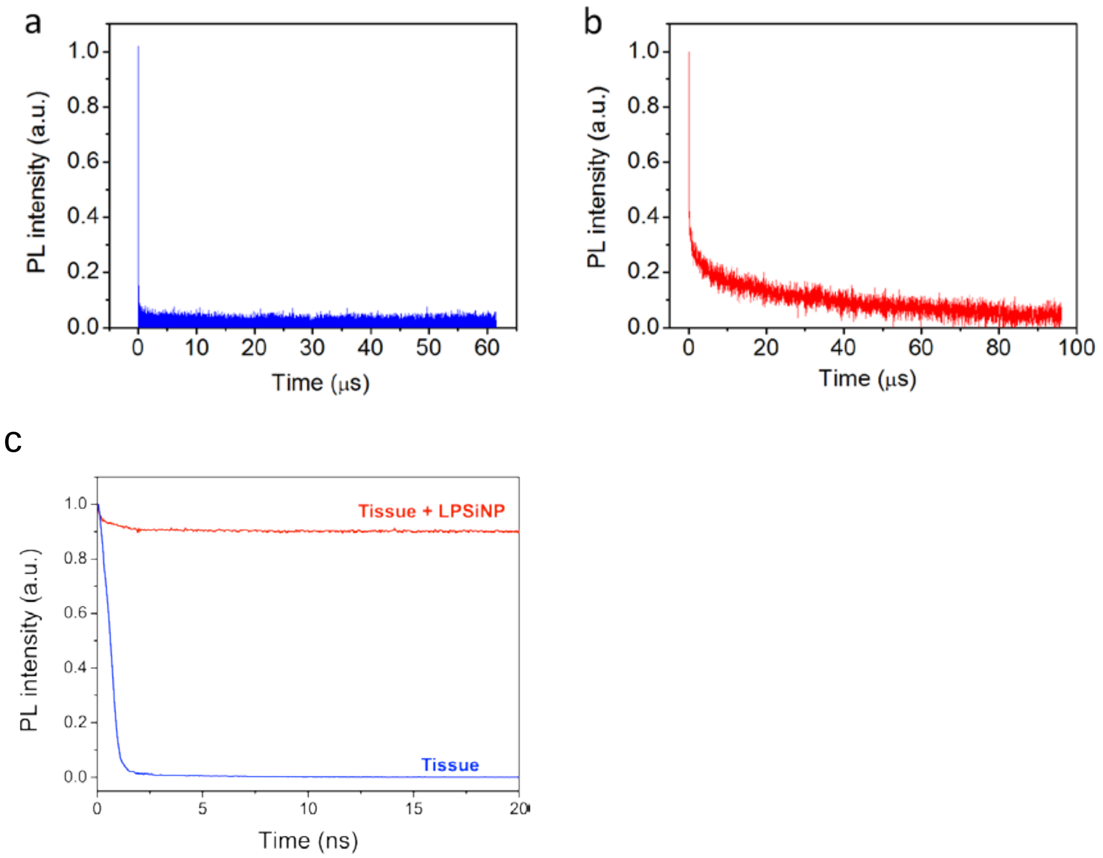

Supplementary Figure S5 | Normalized photoluminescence intensity decay curves *in vivo* (**a, b**) and *in vitro* (**c**) from tissue (autofluorescence, blue traces) and from tissue containing PEG-LPSiNPs (autofluorescence + nanoparticle photoluminescence, red traces). **a**, Decay of intensity of photoluminescence from control mouse tissue (live nude mouse), showing the rapid decay of tissue autofluorescence from the skin. **b**, Decay of intensity of photoluminescence from the same tissue after subcutaneous injection of PEG-LPSiNPs. Decays for **a** and **b** were obtained on a long timescale (microseconds) in order to capture both the short-lived (tissue autofluorescence) and long-lived (PEG-LPSiNPs) signal components. **c,** Normalized photoluminescence intensity decay curves from PEG-LPSiNPs in mouse tissue homogenate, obtained at a shorter time scale (20 nanoseconds). At this shorter timescale the signal from the PEG-LPSiNPs appears nearly constant due to its long excited state lifetime. The short-lived component (< 2 ns) arising from tissue autofluorescence is relatively weak in this sample due to the high concentration of PEG-LPSiNPs. The tissue homogenate sample was isolated from muscle tissue (leg) of a nude mouse and diluted in PBS solution. Spectra from **a** and **b** were obtained using pulsed Nd:YAG laser and a high speed Si photo detector (λ_{ex}: 355 nm, λ_{em}: 400 nm longpass, T = 37°C, 10 Hz repetition rate). Spectra from **c** were obtained using a Horiba Fluorolog spectrofluorometer (λ_{ex}: 470nm, λ_{em}: 700nm, T = 22°C, 10 kHz repetition rate).

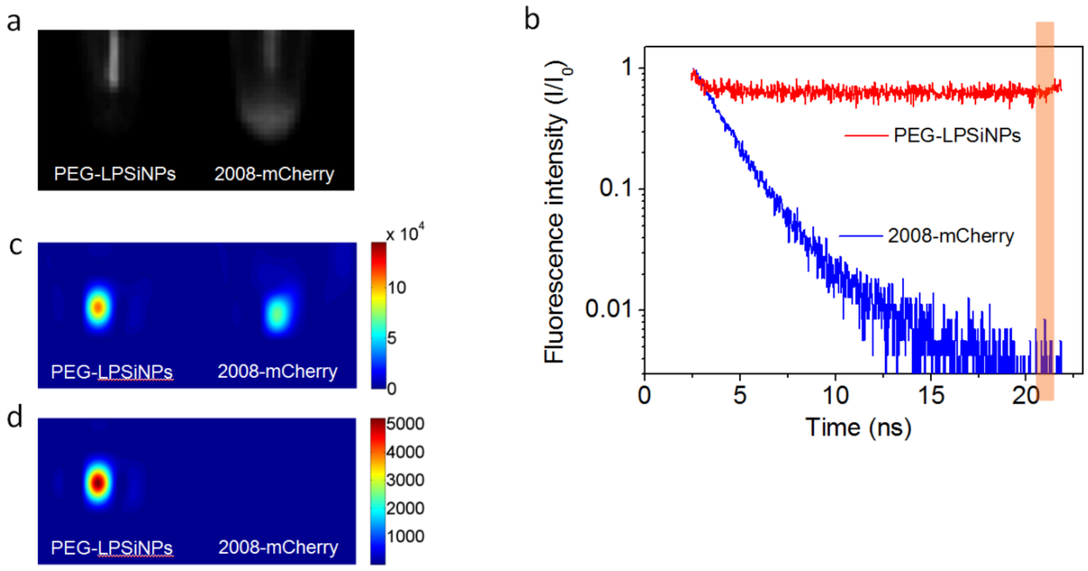

Supplementary Figure S6 | Comparison of PEG-LPSiNPs and mCherry in the time-domain. **a**, Bright filed image of microtubes containing an aqueous dispersion of PEG-LPSiNPs (left, 20 μL, 0.2 mg/mL) or 2008-mCherry cell suspension (right, 20 μL, ~1 million cells). **b**, Fluorescence decay of above samples measured with Optix imaging system. **c**, Continuous wave (CW) fluorescence image of the PEG-LPSiNPs and 2008-mCherry samples in (**a**). **d**, Time-gated (TG) fluorescence image of the PEG-LPSiNPs and 2008-mCherry samples in (**a**).

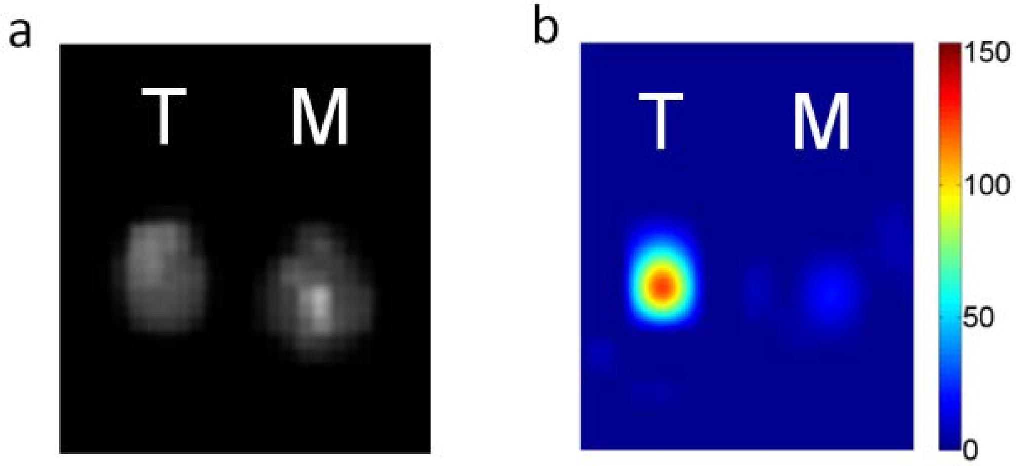

Supplementary Figure S7 | *Ex vivo* fluorescence images of SKOV3 xenograft tumor and muscle in the vicinity of the tumor, harvested from the mouse intravenously injected with PEG-LPSiNPs 24 h post-injection. **a**, Bright field image of the SKOV3 tumor (T) and muscle (M) in the vicinity of the tumor. **b**, Time-gated fluorescence image of the tissues in (**a**).

Etching current density (mA/cm^2)	DLS size (nm)	Surface	Lifetime (μs)		
			2 weeks activation	6 weeks activation	10 weeks activation
50	143	oxide	5.1	6.6	6.9
200	160	oxide	10.0	10.7	11.1
400	159	oxide	12.1	13.2	13.4

Supplementary Table S1. Photoluminescence decay lifetimes of LPSiNPs prepared at different etching current densities and activation durations. The luminescence of porous Si is attributed to a combination of quantum confinement effects and interface defects[43]. Because the porous Si matrix is an assembly of "Si dots" and contains a distribution of interface states, its luminescence decay is multiexponential. Here, we use the time at which the photoluminescence intensity decreases to l/*e* of the initial value after excitation as the average decay lifetime[30,44].

SUPPLEMENTARY REFERENCES

43. Sa'ar, A. Photoluminescence from silicon nanostructures: The mutual role of quantum confinement and surface chemistry. *J. Nanophotonics* 3, 032501 (2009).

44. Xie, Y.H. et al. Luminescence and Structural study of porous silicon films. *J. Appl. Phys.* 71, 2403-2407 (1992).
